# Considerations for the application of polygenic scores to clinical care of individuals with substance use disorders

**DOI:** 10.1172/JCI172882

**Published:** 2024-10-15

**Authors:** Rachel L. Kember, Christal N. Davis, Kyra L. Feuer, Henry R. Kranzler

**Affiliations:** University of Pennsylvania Perelman School of Medicine, Philadelphia, Pennsylvania, USA.

## Abstract

Substance use disorders (SUDs) are highly prevalent and associated with excess morbidity, mortality, and economic costs. Thus, there is considerable interest in the early identification of individuals who may be more susceptible to developing SUDs and in improving personalized treatment decisions for those who have SUDs. SUDs are known to be influenced by both genetic and environmental factors. Polygenic scores (PGSs) provide a single measure of genetic liability that could be used as a biomarker in predicting disease development, progression, and treatment response. Although PGSs are rapidly being integrated into clinical practice, there is little information to guide clinicians in their responsible use and interpretation. In this Review, we discuss the potential benefits and pitfalls of the use of PGSs in the clinical care of SUDs, highlighting current research. We also provide suggestions for important considerations prior to implementing the clinical use of PGSs and recommend future directions for research.

## Background

Substance use disorders (SUDs) are heritable psychiatric conditions characterized by an impaired ability to control substance use despite detrimental consequences, including physiological dependence and tolerance (1). Both environmental and genetic factors influence the development of SUDs. Twin and family studies have estimated the heritability (h^2^) of SUDs to be around 50%, meaning that around half of variation in SUD risk is due to genetic factors (2), with the remaining variability due to environmental factors. Genome-wide association studies (GWAS) are a hypothesis-free method aimed at identifying the genetic variants (e.g., single nucleotide polymorphisms [SNPs]) that account for the variability in risk among populations. Over the past two decades, many GWAS of SUDs have been conducted, successfully identifying multiple genetic variants associated with a variety of substance use traits (Tables 1, 2, 3, and 4). These studies have established that SUDs are complex, polygenic traits, with genetic risk attributable to potentially thousands of genetic variants (3). SNP heritability (h^2^_SNP_), a measure of the proportion of phenotypic variance explained by the common genetic variants measured in GWAS (4), ranges from 1% to 28% for substance use traits, falling short of the estimates produced by twin and family studies (Tables 1–4). This is likely due to some of the current GWAS being underpowered, meaning that not all genetic variants with effects are detected accurately, and due to the contribution of other genetic variants that are not measured in a GWAS (e.g., rare variants or copy number variants). Even if all associated variants are known, for polygenic phenotypes such as SUDs the proportion of phenotypic variance explained by any single variant is very small, meaning that individual variants are ineffective as biomarkers or predictors of disease. Therefore, methods to aggregate the effects of common genetic variants into a single measure that denotes genetic risk for a disease/trait have been developed.

Polygenic scores (PGSs; also known as genetic scores or polygenic risk scores) summarize an individual’s genetic liability for a trait by aggregating the effect sizes of many genetic variants into a single score (5). PGSs are receiving increasing attention as potential biomarkers in a variety of contexts (6). Recently, the FDA approved a genetic risk algorithm comprising 15 candidate genetic variants to predict opioid use disorder (OUD) risk, prompting debate on whether this and other genetic scores (e.g., PGSs) for SUDs are ready for clinical use (7). Such debate stems from the competing potential benefits and pitfalls of using PGSs in the prevention and treatment of SUDs and point to a need to establish guidelines on when PGSs for SUDs should be used in clinical care. For example, PGSs generated from currently available GWAS typically explain only a small proportion of trait variation (usually 2%–10%), which may not translate to clinically significant effects. Furthermore, heritability estimates impose an upper boundary on the ability of genetic risk factors to account for variation in SUDs, presenting a challenge for translating genetic research into clinical practice. However, PGSs capture a larger proportion of genetic liability than single or small groups of variants alone and have been used successfully for medical conditions to identify individuals with disease risk equivalent to monogenic mutations (i.e., those of large effect; ref. 8), predict mortality (9), identify cases with earlier disease onset (10), and provide evidence for cross-trait associations that underlie clinical comorbidities (11). In this Review, we detail current PGS research for substance use traits and explore their potential to enhance the clinical care of individuals with SUDs as well as the challenges and controversies surrounding their use. Finally, we provide suggestions for when, where, and how PGSs should be used in the treatment and prevention of SUDs.

## Calculating PGS

A variety of methods, which differ in their complexity and assumptions, are used to calculate PGSs (12, 13). First, variant effect sizes are estimated in a discovery sample via GWAS of the trait. Second, due to the nonindependence of variants that are close to one another, PGS methods commonly involve either performing clumping to create a set of independent variants or modeling the correlated structure and using this information to adjust the weight of variants. Third, the PGS is calculated in an independent target sample as the sum of the number of variants an individual has, weighted by their effect size, to create a single score for each individual that reflects their genetic liability for that trait. The strength of the association of the PGS with traits can then be evaluated.

## Factors that influence PGS accuracy and utility

Several factors influence the strength of the association of a PGS with a trait. These include the heritability of the trait, the sample size, sex, and genetically inferred ancestral composition of the discovery and target cohorts, the accuracy and depth of phenotyping of the discovery GWAS, and the prevalence of the trait (13).

**Figure 1:**
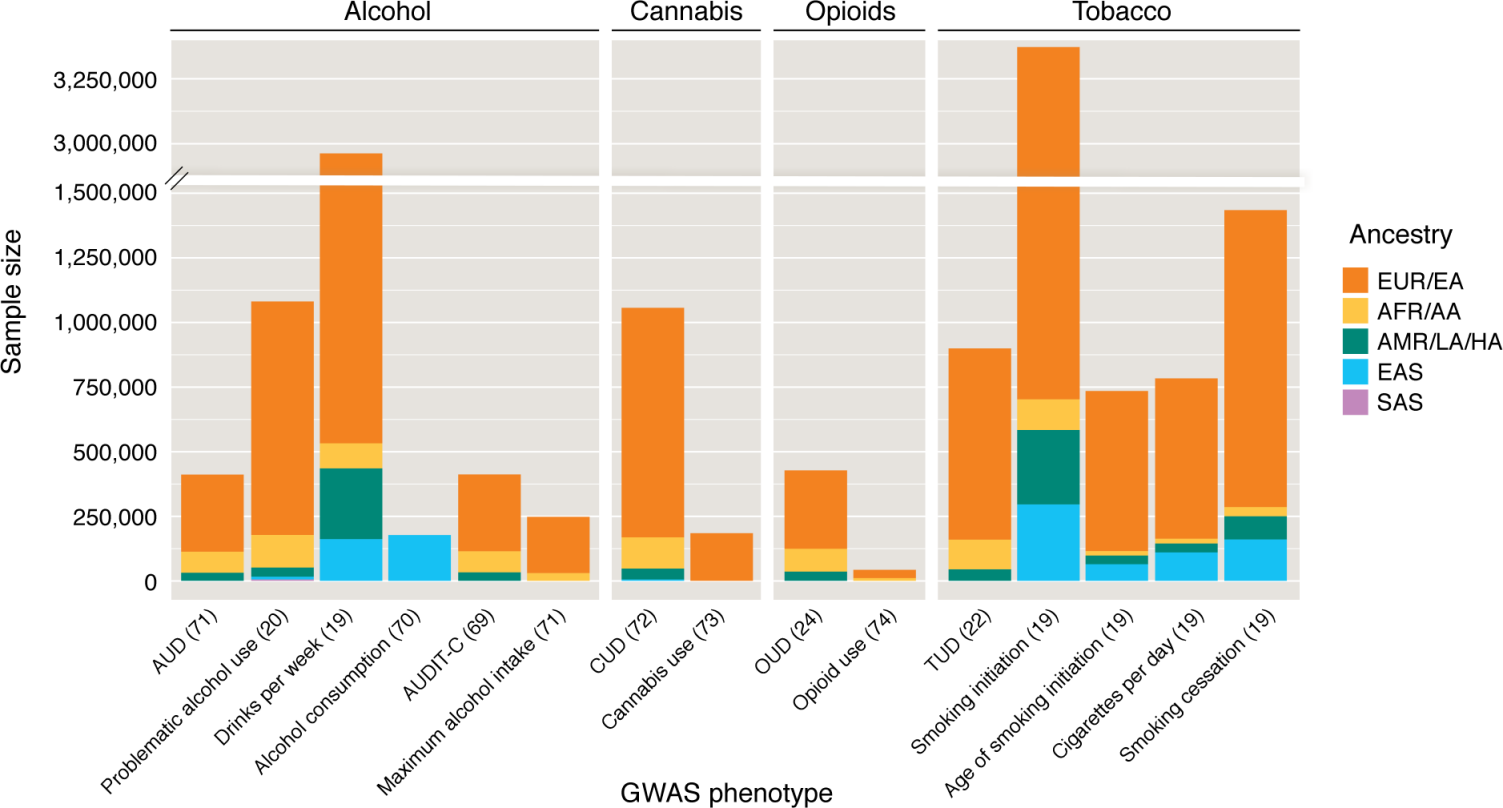
Sample size and ancestry composition of the largest and/or most diverse GWAS of substance use/abuse phenotypes to date. From left to right, bars represent Kember et al., 2023 (69); Zhou et al., 2023 (20); Saunders et al., 2022 (19); Koyanagi et al., 2024 (70); Kember et al., 2023 (69); Deak et al., 2022 (71); Johnson et al., 2020 (72); Pasman et al., 2018 (73); Kember et al., 2022 (24); Polimanti et al., 2020 (74); Toikumo et al., 2024 (22); Saunders et al., 2022 (19); Saunders et al., 2022 (19); Saunders et al., 2022 (19); and Saunders et al., 2022 (19). AUD, alcohol use disorder; AUDIT-C, Alcohol Use Disorders Identification Test–Consumption; CUD, cannabis use disorder; OUD, opioid use disorder; TUD, tobacco use disorder. EUR, European; EA, European American; AFR, African; AA, African American; AMR, admixed American; LA, Latin American; HA, Hispanic American; EAS, East Asian; SAS, South Asian.

The genetic architecture of SUDs affects how well GWAS are able to assess them and therefore how well PGSs perform. SUDs are extremely polygenic in nature, which necessitates very large GWAS sample sizes (upward of ~1 million participants) to acquire enough statistical power to identify SNPs reaching genome-wide significance. While this has been feasible for substances that are legal (e.g., alcohol, tobacco, and, to a lesser extent, cannabis), recruiting users of illicit substances has been more challenging, resulting in underpowered GWAS for these substances. Power is also impacted by the prevalence of the trait in the general population, the discovery cohort, and the target cohort. Another important consideration is the ancestry and sex of the participants of the discovery GWAS and the target cohort. The lack of diversity among GWAS participants remains a major obstacle to the clinical utility of PGS (Figure 1). Variations in genetic architecture and disease prevalence across different ancestral backgrounds all limit the portability of PGSs across populations (14, 15), although the development of transancestry PGS methods has led to improvements in this area (16). Although the past few years have seen substantial increases in the size and ancestral diversity of samples in SUD GWAS (Tables 1–4), this is an area that still requires much improvement. Furthermore, the majority of these studies comprise male individuals, leading to a limited ability to detect sex-specific effects.

One must also consider the ways in which SUDs are defined across cohorts. Recent SUD GWAS efforts have been facilitated by consortia comprising smaller studies performing meta-analyses, such as the Psychiatric Genomics Consortium, and by the use of large electronic health record–based cohorts, such as the United Kingdom Biobank, All of Us, and the Million Veteran Program. Although electronic health record databases have drastically increased the sample sizes and diversity of SUD GWAS, this comes with benefits and trade-offs in the phenotypic information collected, which is generally extracted via International Classification of Diseases billing codes. Electronic health records can be incomplete, contain information from different types of patient interactions/contexts, and may include assessments by clinicians without psychiatric training. However, they also provide benefits for phenotyping, as they gather information across many health domains and provide a more realistic scenario for evaluating precision medicine approaches than highly controlled settings. It is therefore essential that investigators carefully consider their case/control definitions and the strengths and weaknesses of their approach. Because the specificity and power of PGSs depend in part on the phenotype selected (17, 18), it is recommended that one consider how the GWAS phenotype matches the target cohort when generating PGSs.

## Current PGS studies

Despite challenges, PGSs show promise as research tools for substance use traits. Many of the published GWAS listed in Tables 1–4 have been used to calculate PGSs to demonstrate replicability in an independent dataset, accounting for relatively modest proportions of variance. Drinks per week PGS explained 1.2% of the variance in individuals with European ancestry (EUR), translating to a potentially clinically relevant difference of around 3 drinks per week between the bottom and top PGS deciles (19). Alcohol use disorder (AUD) PGS explained 3.3% of the variance in scores on the problem scale of the Alcohol Use Disorders Identification Test (20). Similarly, OUD PGS explained 2.4%–3.8% of the variance in OUD diagnosis (21). The variance explained by tobacco-related PGS has been larger than that of alcohol and opioid PGS. For example, tobacco use disorder (TUD) PGS explained 7.3% of variance in TUD in EUR individuals (22), and smoking initiation PGS was significantly associated with smoking initiation in all ancestral groups, with variance explained ranging from 1% to 9.6% (19). In EUR individuals, 25% of smokers were in the lowest PGS decile compared with 75% in the highest decile, providing meaningful clinical information for those at the ends of the PGS distribution.

PGSs have also been used to explore additional phenotypes associated with genetic liability for the trait or disorder. For instance, PGSs for a substance-related trait (e.g., an exposure measure such as smoking initiation) can also be associated with the disorder (e.g., tobacco dependence) (23). Alternately, PGSs for SUDs have cross-trait associations with common comorbidities, including SUDs other than the primary trait (e.g., association between a problematic alcohol use [PAU] PGS and TUD, ref. 20). Similarly, a TUD PGS was associated with alcohol-related disorders (22), an OUD PGS was associated with TUD (24), and various SUD PGSs have been associated with psychiatric disorders (11, 20–22). SUD PGSs also show associations with somatic traits, implying a shared genetic liability between SUDs and medical disorders. These findings can help elucidate the genetic underpinnings of common SUD comorbidities.

## Clinical utility of PGS

### Disorder development and progression.

PGSs are rapidly being integrated into clinical practice in cardiology, oncology, and other medical diseases because of their potential to enhance precision medicine (25). PGSs show promise for evaluating the risk of disease incidence and progression in several medical conditions, including breast cancer (26), rheumatoid arthritis (27), prostate cancer (28), and coronary disease (29).

PGSs for substance use traits have been evaluated as predictors of the development or clinical progression of both substance use and SUDs in clinically ascertained populations to identify high-risk individuals who might benefit from targeted prevention and intervention efforts. For instance, PGSs for multiple SUDs have shown associations with DSM diagnoses and diagnostic criteria in a sample ascertained for SUDs (23). This sample was also used to explore the association of AUD, OUD, and smoking trajectory PGSs with substance use milestones (i.e., age of onset of use, regular use, problems, and dependence diagnosis) and with progression from regular use to first problems and dependence diagnosis (30). Among EUR individuals, higher AUD, OUD, and smoking trajectory PGSs were associated with earlier onset of their respective substance use milestones but explained only between 0.3% and 2.7% of the variance in outcomes. Among individuals with African ancestry (AFR), the AUD PGS was associated only with the age of onset of regular alcohol use, dependence, and progression from regular use to dependence, and the smoking trajectory PGS predicted only earlier age of initiation. In a different sample, a PGS for age of onset of alcohol dependence explained a statistically significant but modest proportion of the variance (0.03%–0.16%) in alcohol-related measures, including age of onset of intoxication, maximum drinks consumed, and symptom counts (31). Notably, many of these studies have shown modest associations, some of which are not clinically significant.

Despite the small amount of variation explained, PGSs may have clinical utility for individuals at high and low ends of the PGS spectrum. Many current PGS studies for SUDs have reported variance explained only. However, assessing individuals at the extremes of PGSs may provide more information and be a better predictor of risk (32). A PGS for alcohol dependence was associated with a more rapid progression from regular drinking to dependence, an effect that was independent of the age at onset of regular drinking (33). Although only 18.6% of cases had PGS that were at least 1 standard deviation above the sample mean, this may be in part due to ceiling effects, as the sample was enriched for alcohol dependence (34), suggesting that many of these individuals have greater genetic risk than the general population even if their risk is average within this ascertainment sample.

Given that much of the work evaluating the association between PGSs and substance-related milestones has involved samples enriched for these disorders, it is important to evaluate the extent to which the type of cohort and the match between the discovery and target cohorts influences findings. To test this, Savage et al. (2018) used PGS to compare genetic risk prediction of a DSM-IV alcohol dependence criterion count between two population samples and two clinically ascertained samples (35). Within the population samples, PGS generated from one sample were significantly associated with criterion count in the second sample. When the analysis was performed across the sample types (population as discovery and clinical as target, and vice versa), there were no significant associations between the PGS and alcohol problems. Thus, similarity between the discovery and target samples may increase power.

Meta-analyzing across samples of varying compositions may help resolve these issues. Using summary statistics from a GWAS meta-analysis of ascertainment and population-based cohorts (36), Bray and colleagues (37) calculated PGSs in a population-based cohort and a cohort selected to reflect high levels of nicotine dependence. Among the EUR participants in the population-based sample, the PGSs for having ever smoked, early age of initiation, heavier smoking, and cessation were all significantly associated with those traits. Furthermore, in each case the PGS increased the variance accounted for by demographic variables. Similar findings were obtained in the cohort of individuals who smoke, wherein the PGS was significantly associated with nicotine dependence, early age of smoking initiation, heavier smoking, and smoking cessation and also augmented the variance accounted for by demographic measures.

In studies of the general population, greater variation in genetic liability can be leveraged to distinguish levels of genetic risk even when the PGS itself has lower power. For example, in a representative birth cohort in New Zealand, a PGS comprising just 6 genome-wide significant SNPs from a GWAS of smoking quantity (38–40) was examined as a predictor of smoking phenotypes (41). Despite the small number of SNPs and the small discovery dataset, higher PGSs were significantly associated with onset of daily smoking, progression to heavy smoking, persistence of heavy smoking, onset of nicotine dependence, and a failed attempt at smoking cessation. The PGS also predicted smoking risk above and beyond a family history of smoking.

An additional avenue for increasing study power is by focusing on endophenotypes — heritable traits that can clarify the relationship between genetic variations and complex disorders like SUDs. One endophenotype for SUDs is drug metabolism, which is significantly influenced by variation in genes that encode drug-metabolizing enzymes (2) and serves as an intermediate trait between genetic variation and clinical outcomes. Findings from two GWAS meta-analyses of nicotine metabolism (42, 43) were used as discovery samples for creating PGSs in four independent samples (44). Based on evidence of an association between the nicotine-metabolite ratio — a measure of nicotine metabolism and a proxy for CYP2A6 enzyme activity — and smoking behaviors (45), they used 37 significant SNPs identified in the GWAS meta-analyses to calculate PGS in three community-based, cross-sectional samples and one smoking cessation clinical trial. Although the PGS was significantly associated with nicotine metabolism in the target sample, accounting for as much as 16% of the variance in that measure, the PGS did not significantly account for variance in either smoking quantity or the likelihood of smoking cessation. The larger proportion of variance in nicotine metabolism explained by the PGS aligns with the reduced genetic complexity of endophenotypes, which can help to elucidate genetic mechanisms. However, the failure of these scores to predict clinical outcomes suggests that further investigation is needed for endophenotypes to boost the clinical power of PGSs.

### Intervention response and remission.

SUDs are chronic, relapsing conditions, with an annual remission rate of between 6.8% and 9.1% (46). Research is beginning to explore how differences in genetic liability influence SUD treatment response, offering potential pathways to more effective, personalized interventions that may improve remission rates. However, efforts to apply SUD PGSs for precision medicine are limited by the fact that available GWAS are not of treatment outcomes but rather presence or absence of the disorder or a related trait, and existing randomized treatment trials often lack the power to detect pharmacogenetic effects.

Of the various SUDs, TUD is the one for which PGSs may best predict treatment response. Across two randomized controlled trials of EUR individuals attempting to quit smoking, researchers examined the ability of five smoking-related PGSs (i.e., ever smoking, age of smoking initiation, cigarettes per day, smoking persistence, and a combined average of these) to predict outcomes (47). Higher PGSs for a later age of smoking initiation were associated with an increased likelihood of abstinence. Individuals with the highest PGSs had a 45.1% chance of a successful quit attempt, while those with the lowest scores had a 32.8% chance. The combined PGSs (where higher scores indicated greater risk) were associated with lower odds of a successful quit attempt, corresponding to a 15.5% difference in rates of abstinence for those with the highest and lowest PGSs.

The utility of PGSs for predicting remission is more limited for AUD and OUD. For example, researchers sought to predict AUD remission in over 1,300 AFR and EUR individuals using machine learning (48). Remission was defined as no longer meeting DSM-5 criteria for AUD at a follow-up assessment conducted approximately 5 years after the initial assessment. A model including three alcohol-related PGSs (AUD, Alcohol Use Disorders Identification Test – Consumption scores, and maximum alcohol consumption) demonstrated low accuracy at predicting remission (58.6%). A PGS derived using findings from a GWAS meta-analysis of time until relapse following pharmacological treatment for AUD (49) accounted for a small proportion of variance (1.3%) in treatment outcomes in a holdout sample. Similarly, genetic variants associated with several OUD outcomes (e.g., continued use, relapse, methadone dose, and overdose) accounted for a very small amount of the variance in methadone dose (3.45 × 10^–3^%) in an independent sample (50).

Although research is lacking on the utility of PGS for predicting cannabis use disorder (CUD) treatment outcomes, a longitudinal preventive intervention study of over 600 youth investigated whether a smoking cessation PGS interacted with a classroom behavior management intervention to influence time to cannabis initiation (51). There was a significant PGS-by-intervention interaction, such that children with a high PGS (i.e., greater likelihood of smoking cessation) benefited the most and had the lowest cannabis initiation rates by age 18. Although not an intervention for CUD specifically, this study highlights the potential utility of SUD-related PGSs for predicting cannabis intervention responses.

### Do PGSs provide added clinical utility?

To be of clinical utility, PGSs should demonstrate incremental predictive value for SUD-related outcomes beyond known relevant environmental risk factors. Although genotyping costs have decreased substantially, the cost and complexity of PGSs may not be warranted if phenotypic characteristics sufficiently capture SUD-related risk. Studies have shown that such characteristics can help to predict SUDs. For example, in over 600 adolescents assessed from ages 16 to 25 years, a transmissible liability index comprising both phenotypic features that distinguish children of SUD-affected and SUD-unaffected parents and measures of substance use predicted OUD at age 25 with 86% accuracy (52). PGSs that augment that predictive ability would justify their inclusion in a predictive model.

Several studies have examined the contribution of PGSs to SUD-related outcomes after considering other risk factors. Four longitudinal cohorts were leveraged to examine whether a clinical/environmental risk index and PGS predicted alcohol, nicotine, or any substance dependence in young adulthood (53). The environmental index included measures of socioeconomic status (SES), family history of SUDs, childhood internalizing/externalizing symptoms, trauma exposure, and adolescent personal and peer substance use. Adding six PGSs for substance use and related phenotypes (i.e., externalizing problems, depression, PAU, drinks per week, cigarettes per day, and schizophrenia) explained minimal variance in outcomes. Although PGSs remained significant predictors, most of the explanatory power was due to the environmental index. Similarly, in tobacco cessation trials, adding PGSs for smoking behaviors to a model with clinical predictors significantly increased the area under the curve (AUC), but the magnitude of the change was small (AUC = 0.01), whereas basic clinical predictors (e.g., cigarettes per day and treatment type) had a greater effect on model performance (AUC = 0.05). Assessing phenotypes that index those captured by PGSs (e.g., asking about adolescent alcohol use rather than calculating a PGS for alcohol consumption) may provide a better estimate of an individual’s risk.

Other studies argue that the fact that PGSs remain significant predictors after inclusion of clinical risk factors highlights the unique information these scores provide, even if the variance explained by them is low. For example, a longitudinal study of a sample enriched for parental AUD (54) found that a PAU PGS significantly predicted alcohol-related problems in young adulthood after accounting for demographics, parental history of AUD, and adolescent alcohol use and problems. The significance of the PGS in the adjusted model suggests that it captures information distinct from family and personal histories of substance use.

Within-family analyses may provide greater insight into these associations by accounting for indirect genetic effects, which include the influence of an individual’s genes on their environment (and in turn their behaviors) as well as the effects of parents’ genotypes on the family environment and child’s phenotype, even when the specific genes are not inherited by the child. Twin studies, by comparing siblings who share varying degrees of genetic similarity, help to disentangle indirect from direct genetic influences. In a longitudinal twin study that included six PGSs for alcohol, nicotine, and cannabis use and use disorder, the PGSs almost always remained significant predictors of future substance use after controlling for comorbid SUDs and family history (55). However, in dizygotic cotwin comparisons, which more fully account for familial factors, many PGS effects were not significant. This underscores the complex etiology of SUDs and indicates that genetic predisposition (as assessed by between-family PGS) reflects a combination of direct and indirect genetic effects.

PGSs may provide distinct information in prediction models by examining gene-environment interplay. For example, gene-environment interactions help identify who is most vulnerable to environmental risk factors, and gene-environment correlations indicate how genetic predispositions shape environmental exposures. Among Dutch twins and their family members, an alcohol consumption PGS interacted with SES, such that the PGS was associated with higher levels of alcohol use only among those with higher SES (56). Other research using Australian twins found a gene-environment correlation with a similar direction of effect, such that a higher educational attainment PGS was correlated with an increased likelihood of adolescent alcohol use (57). Thus, not only are genetic and environmental factors independently associated with substance use and use disorders, but their interplay may also provide unique insights.

### Barriers and considerations for implementation.

Although there is potential for PGSs to enhance health care, several barriers warrant consideration before PGS can be incorporated into the standard clinical care of individuals with SUDs. There is also the added complication that unlike physical diseases, for which PGSs currently show utility, SUD diagnoses are stigmatized (58). Thus, the implementation of PGSs for SUDs in clinical settings necessitates an evaluation of their utility and a consideration of factors, including ethical concerns, that could hinder their use. These factors impact when and where to use PGSs, which PGSs to use, and whether the PGS provides useful information.

First, the clinician must consider the cohort in which they are applying PGSs. If applying in a general population with average risk of developing disorder, then the PGS will identify those at higher risk of developing the disorder in their lifetime. These individuals could be encouraged to reduce their exposure to substances or other environmental factors in order to reduce their overall risk. If applying in a set of individuals with symptoms of the disorder, the PGS could help identify individuals who are at higher likelihood of an increase in symptoms. In a set of individuals with the disorder, the PGS could identify those who may best respond to particular types of treatment.

One advantage of PGSs compared with environmental risk factors is that they can be measured prior to the development of any symptoms. However, most GWAS are cross-sectional, making it difficult to evaluate potential differences in the effects of genetic liability across development. Before applying PGSs clinically, it is important to understand how such differences might affect their performance. For example, one study evaluated the extent to which genetic influences on alcohol use frequency were common or unique to development (59). Although the sample was small, there was preliminary evidence that an age-specific PGSs better predicted adult alcohol use frequency than a PGS of genetic influences across development. A study applying time-varying effects models further supported age-specific genetic effects, as a PGS for alcohol consumption was associated with alcohol use in young adulthood but not adolescence (60). Another study found that an externalizing PGS (comprising antisocial behavior, attention deficit hyperactivity disorder, cannabis use, and alcohol dependence) did not predict externalizing or internalizing behaviors in older adults (61), suggesting that other genetic or environmental risk factors may become more important for understanding liability for psychopathology as individuals age. This variability in performance across development could pose limitations for the clinical applications of PGSs. Longitudinal, developmental datasets, like the Adolescent Brain Cognitive Development study, present an opportunity to address these questions in the future as the cohort ages into substance use.

The next consideration for the clinician is which PGS to use. GWAS have been conducted for SUDs, but also for exposure to substances, and for broader phenotypes that may denote general risk such as externalizing behaviors. Many current PGSs are not specific for the disorder or outcome of interest, meaning that high scores could be indicative of any number of outcomes. Reflecting their shared etiology, PGSs for multiple SUDs are associated with lifetime opioid misuse (62), with little specificity to the substance for which they were derived. Even non-SUD PGSs (i.e., those for schizophrenia, bipolar disorder, and major depression) show considerable overlap with SUDs (63). Depending on the use case for the PGS, this may be a concern. For instance, if the PGS is used to discriminate between diagnoses, specificity is required. However, if the PGS is used to predict a single outcome, then the main concern will be the strength of the association with that phenotype, regardless of others. Enhancing PGS specificity using deeper, symptom-level phenotyping and techniques like genomic structural equation modeling, which can distinguish shared and disorder-specific effects, may help develop more clinically useful PGSs. Another current limitation of PGSs is that they can lack stability in an individual across different discovery GWAS for the same phenotype. While PGSs for the same trait were found to be highly correlated at the population level, PGSs for different discovery GWAS have only modest correlation at the individual level, with overlap for patients in the top quantiles based on different GWAS for the same trait being as low as 20% (64).

Finally, the clinician must decide whether the PGS provides information that is useful for the patient. If receiving PGS results does not change behavior or if results are misunderstood or negatively perceived by patients, they may have no or limited clinical utility. To evaluate this, several studies have provided genetic risk results for tobacco-related diseases to individuals who smoke (65–67). Across these studies, individuals who smoke expressed interest in receiving personalized risk scores, had high recall for the information provided, and often reduced smoking following the intervention. Research for other SUDs is more limited, but in one study receipt of a high hypothetical PGS for AUD was associated with greater psychological distress (68), though participants reported that they would be more likely to talk to a health care provider about their risk and reduce their alcohol use as scores increased. Studies of PGSs for tobacco use contradict these findings, finding that participants appreciated receiving the genetic results and did not show increased anxiety or depression after receiving high risk results (66). While the potential for increased distress alongside the potential for positive health change needs to be carefully weighed by patients and providers prior to SUD-related genetic testing, findings for tobacco use are promising.

## Future directions

**Figure 2:**
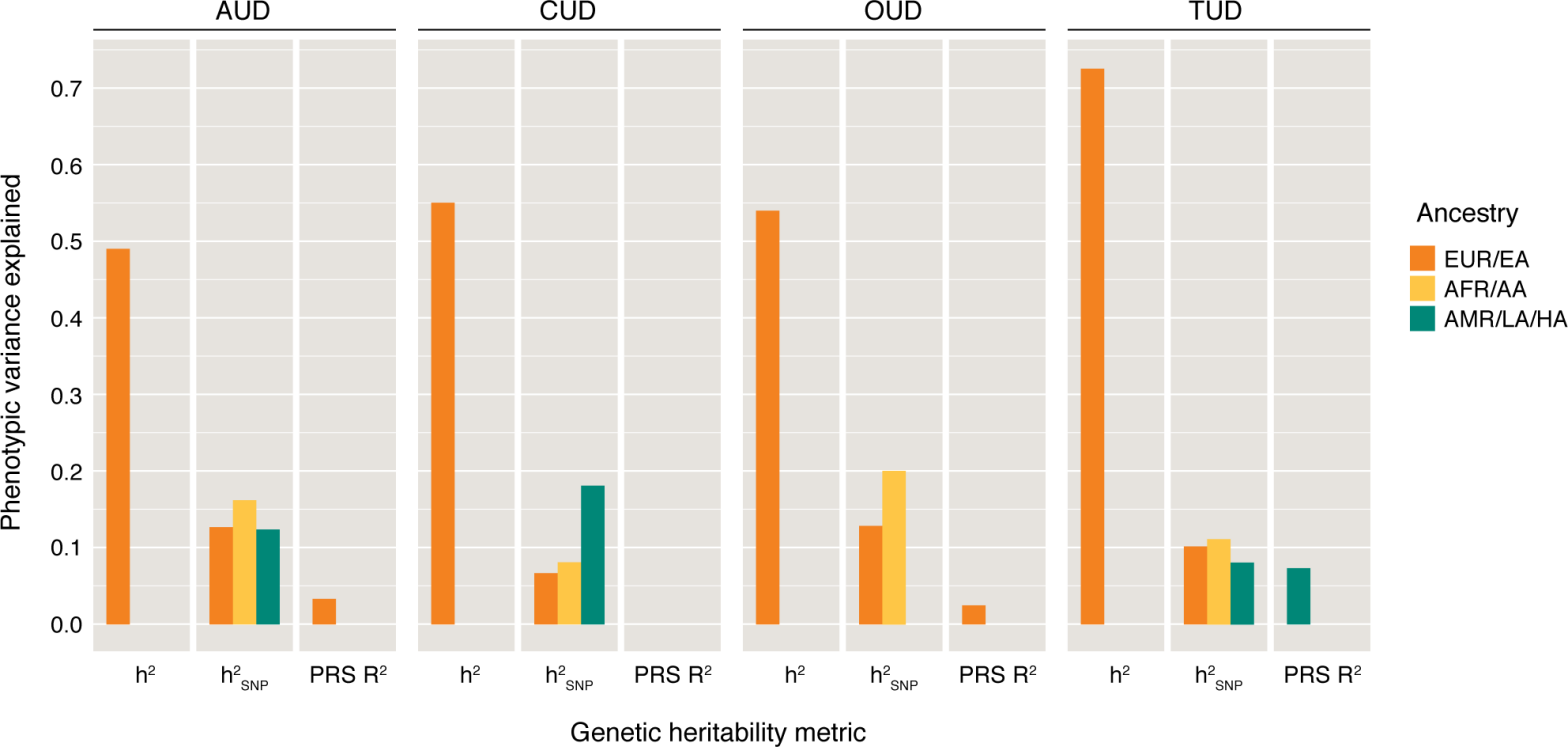
Family-based and SNP-based heritability estimates and variance explained by polygenic scores for substance use disorders. Absence of bars indicates that these data are not available for the corresponding ancestry group. Heritability estimates are on the liability scale. Family-based (h^2^) estimates are derived, from left to right, from Verhulst, Neale, and Kendler, 2015 (75); Verweij et al., 2010 (76); Tsuang et al., 1998 (77); and Do et al., 2015 (78). SNP-based (h2_SNP_) estimates are derived, from left to right, from Zhou et al., 2023 (20); Zhou et al., 2023 (20); Zhou et al., 2023 (20); Levey et al., 2023 (79); Levey, et al., 2023 (79); Levey et al., 2023 (79); Deak et al., 2022 (21); Kember et al., 2022 (24); Toikumo et al., 2024 (22); Toikumo et al., 2024 (22); and Toikumo et al., 2024 (22). PRS polygenic scores (R^2^) estimates are derived, from left to right, from Zhou et al., 2023 (20); Johnson et al., 2020 (72); Deak et al., 2022 (21); and Toikumo et al., 2024 (22). AUD, alcohol use disorder; CUD, cannabis use disorder; OUD, opioid use disorder; TUD, tobacco use disorder; EUR, European; EA, European American; AFR, African; AA, African American; AMR, Admixed American; LA, Latin American; HA, Hispanic American.

While PGSs have more predictive utility than single genetic variants, the variance and heritability explained by these scores for highly polygenic disorders are typically low compared with family-study–based estimates (Figure 2). The “missing heritability” that is currently not captured by variant-based PGS is likely is due in part to the complex genetic architecture of polygenic disorders and epistatic (i.e., gene × gene interactions) effects. The overwhelming majority of GWAS interrogate associations of disease with common variant (minor allele frequency >1%) via array genotyping. This does not account for other types of common and rare variation (for example, copy number variants, chromosomal translations, and insertions and deletions), which may contribute to SUD risk. As sequencing technologies improve and the cost of whole-genome sequencing decreases, it will become increasingly common to augment PGSs with additional types of variation to assess risk across the diverse genetic architecture of SUDs. PGSs also do not provide insights into the biology underlying an individual’s unique risk for an SUD. While the fact that PGSs are derived from DNA can be advantageous for clinical purposes (as PGSs can be ascertained at any time from a saliva or blood sample), the static nature of PGSs means that the contexts in which these variants act (e.g., point in time, cell type) are unknown. This is compounded by the cross-sectional nature of GWAS, which means that the PGSs derived from them do not indicate at which time point these variants are biologically impactful and when intervention would be most beneficial. An active area of research that addresses these concerns aims to develop methods for “pathway” PGSs, which assign variants to biological pathways and calculate a pathway-specific PGS. This approach could provide a more granular assessment of disease risk by partitioning individuals into groups based on similar biological profiles, potentially allowing the identification of medications that may be particularly beneficial for certain patient groups.

## Conclusions

Identifying biomarkers that are effective predictors of SUD development, progression, and treatment response would advance precision medicine by improving diagnosis and treatment. PGSs are one such tool that may help to realize this goal, but prior to implementation, improvements in PGSs are needed to ensure that the information provided to patients is accurate, equitable, reliable, and useful. The value of PGSs lies not in replacing existing behavioral and clinical predictors but in complementing them. PGSs may be particularly useful in cases in which phenotypic data are incomplete, unavailable, or ambiguous, with diminishing returns when more extensive behavioral and clinical data are available. It is important to evaluate PGSs alongside other risk factors in a holistic manner, considering the strengths and limitations of each.

## Figures and Tables

**Table 4:**
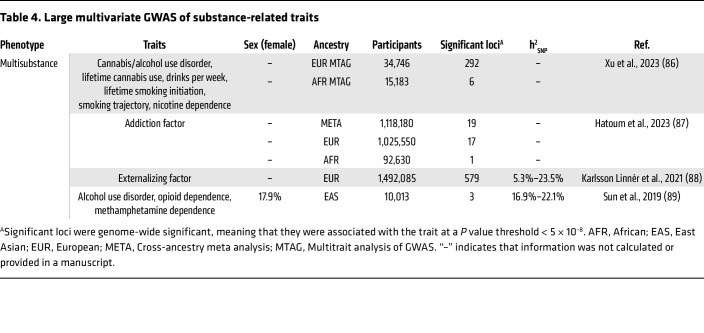
Large multivariate GWAS of substance-related traits

**Table 3:**
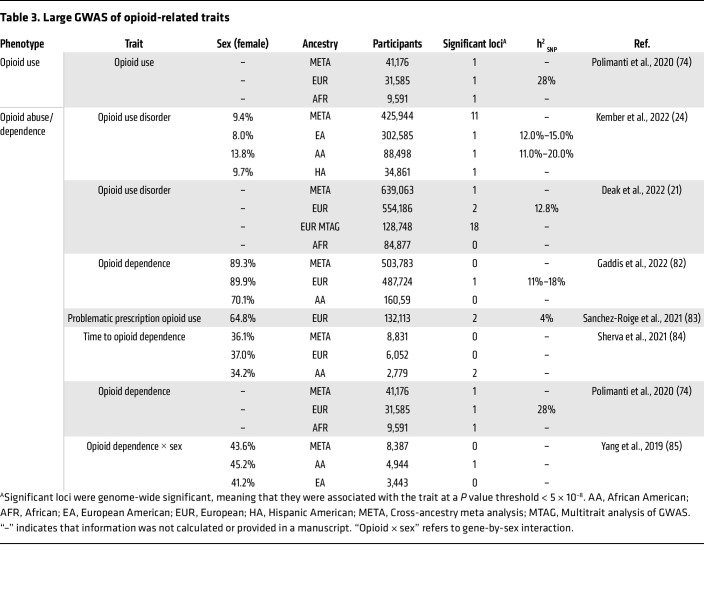
Large GWAS of opioid-related traits

**Table 2:**
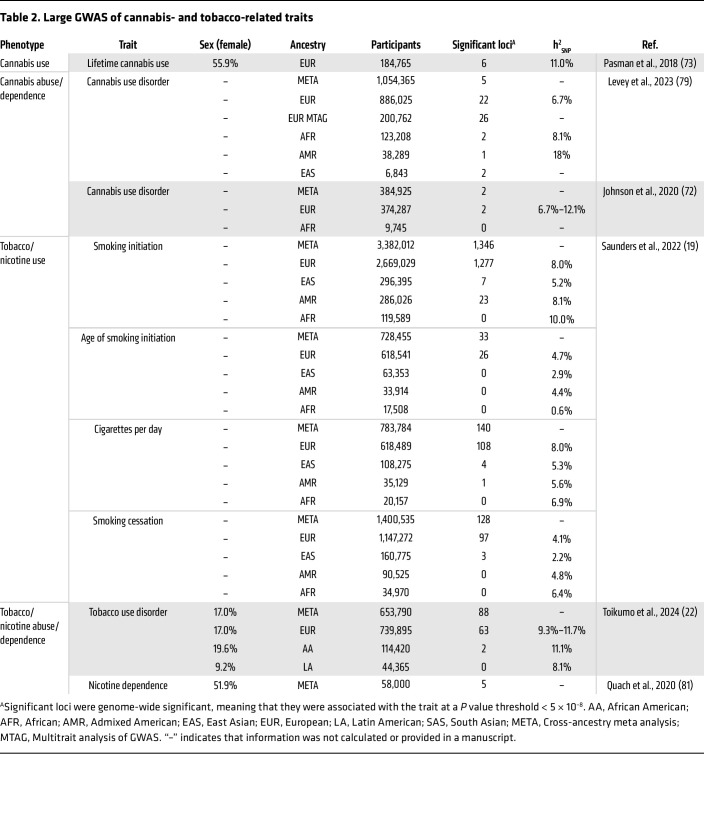
Large GWAS of cannabis- and tobacco-related traits

**Table 1:**
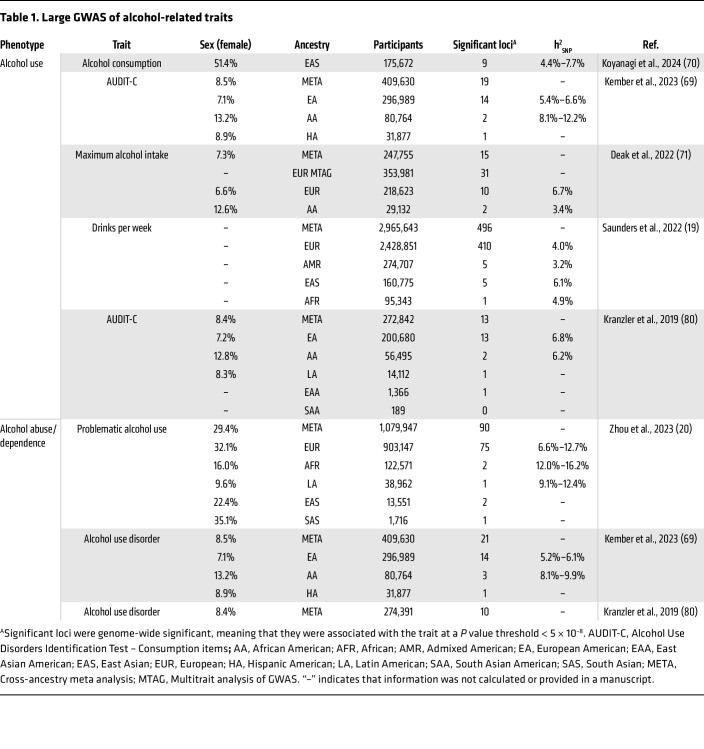
Large GWAS of alcohol-related traits

## Version 1 10/15/2024

Electronic publication

## References

[2] Deak JD, Johnson EC (2021). Genetics of substance use disorders: a review. Psychol Med.

[3] Watanabe K (2019). A global overview of pleiotropy and genetic architecture in complex traits. Nat Genet.

[4] Zhu H, Zhou X (2020). Statistical methods for SNP heritability estimation and partition: A review. Comput Struct Biotechnol J.

[5] Torkamani A (2018). The personal and clinical utility of polygenic risk scores. Nat Rev Genet.

[6] Lewis CM, Vassos E (2020). Polygenic risk scores: from research tools to clinical instruments. Genome Med.

[7] https://www.washingtonpost.com/health/2024/04/05/avertd-fda-opioid-addiction-test/.

[8] Khera AV (2018). Genome-wide polygenic scores for common diseases identify individuals with risk equivalent to monogenic mutations. Nat Genet.

[9] Levin MG (2018). Genomic risk stratification predicts all-cause mortality after cardiac catheterization. Circ Genom Precis Med.

[10] Musliner KL (2019). Association of polygenic liabilities for major depression, bipolar disorder, and schizophrenia with risk for depression in the Danish population. JAMA Psychiatry.

[11] Hartwell EE (2022). Genetic liability for substance use associated with medical comorbidities in electronic health records of African- and European-ancestry individuals. Addict Biol.

[12] Ni G (2021). A comparison of ten polygenic score methods for psychiatric disorders applied across multiple cohorts. Biol Psychiatry.

[13] Ma Y, Zhou X (2021). Genetic prediction of complex traits with polygenic scores: a statistical review. Trends Genet.

[14] Duncan L (2019). Analysis of polygenic risk score usage and performance in diverse human populations. Nat Commun.

[15] Martin AR (2019). Clinical use of current polygenic risk scores may exacerbate health disparities. Nat Genet.

[16] Kachuri L (2024). Principles and methods for transferring polygenic risk scores across global populations. Nat Rev Genet.

[17] Dahl A (2023). Phenotype integration improves power and preserves specificity in biobank-based genetic studies of major depressive disorder. Nat Genet.

[18] Cai N (2020). Minimal phenotyping yields genome-wide association signals of low specificity for major depression. Nat Genet.

[19] Saunders GRB (2022). Genetic diversity fuels gene discovery for tobacco and alcohol use. Nature.

[20] Zhou H (2023). Multi-ancestry study of the genetics of problematic alcohol use in over 1 million individuals. Nat Med.

[21] Deak JD (2022). Genome-wide association study in individuals of European and African ancestry and multi-trait analysis of opioid use disorder identifies 19 independent genome-wide significant risk loci. Mol Psychiatry.

[22] Toikumo S (2024). Multi-ancestry meta-analysis of tobacco use disorder identifies 461 potential risk genes and reveals associations with multiple health outcomes. Nat Hum Behav.

[23] Kember RL (2023). Phenome-wide association analysis of substance use disorders in a deeply phenotyped sample. Biol Psychiatry.

[24] Kember RL (2022). Cross-ancestry meta-analysis of opioid use disorder uncovers novel loci with predominant effects in brain regions associated with addiction. Nat Neurosci.

[25] Denny JC, Collins FS (2021). Precision medicine in 2030-seven ways to transform healthcare. Cell.

[26] Gao C (2021). Risk of breast cancer among carriers of pathogenic variants in breast cancer predisposition genes varies by polygenic risk score. J Clin Oncol.

[27] Honda S (2022). Association of polygenic risk scores with radiographic progression in patients with rheumatoid arthritis. Arthritis Rheumatol.

[28] Lim AJW (2023). Robust SNP-based prediction of rheumatoid arthritis through machine-learning-optimized polygenic risk score. J Transl Med.

[29] Sandhu Roopinder K (2022). Polygenic risk score predicts sudden death in patients with coronary disease and preserved systolic function. J Am Coll Cardiol.

[30] Kranzler HR (2023). Does polygenic risk for substance-related traits predict ages of onset and progression of symptoms?. Addiction.

[31] Kapoor M (2016). Genome-wide polygenic scores for age at onset of alcohol dependence and association with alcohol-related measures. Transl Psychiatry.

[32] Nurnberger JI (2022). High polygenic risk scores are associated with early age of onset of alcohol use disorder in adolescents and young adults at risk. Biol Psychiatry Glob Open Sci.

[33] Yeung EW (2022). Effects of genetic risk for alcohol dependence and onset of regular drinking on the progression to alcohol dependence: A polygenic risk score approach. Drug Alcohol Depend.

[34] Ehlers CL (2010). Age at regular drinking, clinical course, and heritability of alcohol dependence in the San Francisco family study: a gender analysis. Am J Addict.

[35] Savage JE (2018). Polygenic risk score prediction of alcohol dependence symptoms across population-based and clinically ascertained samples. Alcohol Clin Exp Res.

[36] Liu M (2019). Association studies of up to 1.2 million individuals yield new insights into the genetic etiology of tobacco and alcohol use. Nat Genet.

[37] Bray MJ (2021). Studying the utility of using genetics to predict smoking-related outcomes in a population-based study and a selected cohort. Nicotine Tob Res.

[38] Thorgeirsson TE (2010). Sequence variants at CHRNB3-CHRNA6 and CYP2A6 affect smoking behavior. Nat Genet.

[39] Liu JZ (2010). Meta-analysis and imputation refines the association of 15q25 with smoking quantity. Nat Genet.

[40] Furberg H (2010). Genome-wide meta-analyses identify multiple loci associated with smoking behavior. Nat Genet.

[41] Belsky DW (2013). Polygenic risk and the developmental progression to heavy, persistent smoking and nicotine dependence: evidence from a 4-decade longitudinal study. JAMA Psychiatry.

[42] Loukola A (2015). A genome-wide association study of a biomarker of nicotine metabolism. PLoS Genet.

[43] Baurley JW (2016). Genome-wide association of the laboratory-based nicotine metabolite ratio in three ancestries. Nicotine Tob Res.

[44] Chen LS (2018). Use of polygenic risk scores of nicotine metabolism in predicting smoking behaviors. Pharmacogenomics.

[45] Siegel SD (2020). The use of the nicotine metabolite ratio as a biomarker to personalize smoking cessation treatment: current evidence and future directions. Cancer Prev Res (Phila).

[46] Fleury MJ (2016). Remission from substance use disorders: a systematic review and meta-analysis. Drug Alcohol Depend.

[47] Bray M (2022). The promise of polygenic risk prediction in smoking cessation: evidence from two treatment trials. Nicotine Tob Res.

[48] Kinreich S (2021). Predicting alcohol use disorder remission: a longitudinal multimodal multi-featured machine learning approach. Transl Psychiatry.

[49] Biernacka JM (2021). Genetic contributions to alcohol use disorder treatment outcomes: a genome-wide pharmacogenomics study. Neuropsychopharmacology.

[50] McEvoy A (2023). A genome-wide association, polygenic risk score and sex study on opioid use disorder treatment outcomes. Sci Rep.

[51] Musci RJ (2018). Polygenic score × intervention moderation: an application of discrete-time survival analysis to model the timing of first marijuana use among urban youth. Prev Sci.

[52] Tarter RE (2020). Forecasting opioid use disorder at 25 years of age in 16-year-old adolescents. J Pediatr.

[53] Barr PB (2022). Clinical, environmental, and genetic risk factors for substance use disorders: characterizing combined effects across multiple cohorts. Mol Psychiatry.

[54] Wang FL (2023). Polygenic risk score for problematic alcohol use predicts heavy drinking and alcohol use disorder symptoms in young adulthood after accounting for adolescent alcohol use and parental alcohol use disorder. Drug Alcohol Depend.

[55] Schaefer JD (2023). Associations between polygenic risk of substance use and use disorder and alcohol, cannabis, and nicotine use in adolescence and young adulthood in a longitudinal twin study. Psychol Med.

[56] Pasman JA (2020). Substance use: interplay between polygenic risk and neighborhood environment. Drug Alcohol Depend.

[57] Davis CN (2022). Educational attainment polygenic scores: examining evidence for gene-environment interplay with adolescent alcohol, tobacco and cannabis use. Twin Res Hum Genet.

[58] van Boekel LC (2013). Stigma among health professionals towards patients with substance use disorders and its consequences for healthcare delivery: systematic review. Drug Alcohol Depend.

[59] Thomas NS (2024). A developmentally-informative genome-wide association study of alcohol use frequency. Behav Genet.

[60] Elam KK (2021). Age varying polygenic effects on alcohol use in African Americans and European Americans from adolescence to adulthood. Sci Rep.

[61] Gard AM (2021). Phenotypic and genetic markers of psychopathology in a population-based sample of older adults. Transl Psychiatry.

[62] Rabinowitz JA (2022). Positive associations between cannabis and alcohol use polygenic risk scores and phenotypic opioid misuse among African-Americans. PLoS One.

[63] Rodrigue AL (2023). Specificity of psychiatric polygenic risk scores and their effects on associated risk phenotypes. Biol Psychiatry Glob Open Sci.

[64] Schultz LM (2022). Stability of polygenic scores across discovery genome-wide association studies. HGG Adv.

[65] Ramsey AT (2021). Proof of concept of a personalized genetic risk tool to promote smoking cessation: high acceptability and reduced cigarette smoking. Cancer Prev Res (Phila).

[66] Hartz SM (2015). Return of individual genetic results in a high-risk sample: enthusiasm and positive behavioral change. Genet Med.

[67] Olfson E (2016). Implications of personal genomic testing for health behaviors: the case of smoking. Nicotine Tob Res.

[68] Driver MN (2023). The impact of receiving polygenic risk scores for alcohol use disorder on psychological distress, risk perception, and intentions to reduce drinking. Am J Med Genet B Neuropsychiatr Genet.

[69] Kember RL (2023). Genetic underpinnings of the transition from alcohol consumption to alcohol use disorder: shared and unique genetic architectures in a cross-ancestry sample. Am J Psychiatry.

[70] Koyanagi YN (2024). Genetic architecture of alcohol consumption identified by a genotype-stratified GWAS and impact on esophageal cancer risk in Japanese people. Sci Adv.

[71] Deak JD (2022). Genome-wide investigation of maximum habitual alcohol intake in US veterans in relation to alcohol consumption traits and alcohol use disorder. JAMA Netw Open.

[72] Johnson EC (2020). A large-scale genome-wide association study meta-analysis of cannabis use disorder. Lancet Psychiatry.

[73] Pasman JA (2018). GWAS of lifetime cannabis use reveals new risk loci, genetic overlap with psychiatric traits, and a causal influence of schizophrenia. Nat Neurosci.

[74] Polimanti R (2020). Leveraging genome-wide data to investigate differences between opioid use vs. opioid dependence in 41,176 individuals from the Psychiatric Genomics Consortium. Mol Psychiatry.

[75] Verhulst B (2015). The heritability of alcohol use disorders: a meta-analysis of twin and adoption studies. Psychol Med.

[76] Verweij KJH (2010). Genetic and environmental influences on cannabis use initiation and problematic use: a meta-analysis of twin studies. Addiction.

[77] Tsuang MT (1998). Co-occurrence of abuse of different drugs in men: the role of drug-specific and shared vulnerabilities. Arch Gen Psychiatry.

[78] Do EK (2015). Genetic and environmental influences on smoking behavior across adolescence and young adulthood in the Virginia twin study of adolescent behavioral development and the transitions to substance abuse follow-up. Twin Res Hum Genet.

[79] Levey DF (2023). Multi-ancestry genome-wide association study of cannabis use disorder yields insight into disease biology and public health implications. Nat Genet.

[80] Kranzler HR (2019). Genome-wide association study of alcohol consumption and use disorder in 274,424 individuals from multiple populations. Nat Commun.

[81] Quach BC (2020). Expanding the genetic architecture of nicotine dependence and its shared genetics with multiple traits. Nat Commun.

[82] Gaddis N (2022). Multi-trait genome-wide association study of opioid addiction: OPRM1 and beyond. Sci Rep.

[83] Sanchez-Roige S (2021). Genome-wide association study of problematic opioid prescription use in 132,113 23andMe research participants of European ancestry. Mol Psychiatry.

[84] Sherva R (2021). Genome-wide association study of phenotypes measuring progression from first cocaine or opioid use to dependence reveals novel risk genes. Explor Med.

[85] Yang B-Z (2019). Genomewide gene-by-sex interaction scans identify ADGRV1 for sex differences in opioid dependent African Americans. Sci Rep.

[86] Xu H (2023). Identifying genetic loci and phenomic associations of substance use traits: a multi-trait analysis of GWAS (MTAG) study. Addiction.

[87] Hatoum AS (2023). Multivariate genome-wide association meta-analysis of over 1 million subjects identifies loci underlying multiple substance use disorders. Nat Ment Health.

[88] Karlsson Linnér R (2021). Multivariate analysis of 1.5 million people identifies genetic associations with traits related to self-regulation and addiction. Nat Neurosci.

[89] Sun Y (2021). Identification of novel risk loci with shared effects on alcoholism, heroin, and methamphetamine dependence. Mol Psychiatry.

